# Incorporation of ifosfamide into various essential oils -based nanoemulsions ameliorates its apoptotic effect in the cancers cells

**DOI:** 10.1038/s41598-018-37048-x

**Published:** 2019-01-24

**Authors:** Mayson H. Alkhatib, Sahar M. AlMotwaa, Huda M. Alkreathy

**Affiliations:** 1 Department of Biochemistry, Faculty of Science, King Abdulaziz University, Jeddah, Saudi Arabia; 2 Chemistry Department, College of Science and Humanities, Shaqra University, Shagra, Saudi Arabia; 3 Department of Pharmacology, Faculty of Medicine, King Abdulaziz University, Jeddah, Saudi Arabia

## Abstract

The chemotherapeutic drugs, loaded in nanocarriers, have recently attracted the pharmaceutical industries due to their limited adverse side effects. The objective of the current study was to incorporate the ifosfamide (IFO) into two different essential oils-based nanoemulsions, lemon (LEM-IFO) and salvia (SAL-IFO). The antiproliferation activities of the resulted formulas were evaluated in the MCF-7 breast cancer cells and HeLa cervical cancers cells. The cytotoxic effect of the NE formulas was detected by the MTT assay, DAPI stain and light microscopy. The z-average diameters range of LEM-IFO and SAL-IFO, determined by the zetasizer, were 49.15–61.81 nm and 56.64–64.62 nm, respectively. The half maximal inhibitory concentration (IC_50_) of LEM-IFO and SAL-IFO, applied into the HeLa cells, were 0.165 ± 0.025 and 0.141 ± 0.035 mM, respectively, whereas the IC_50_ of LEM-IFO and SAL-IFO subjected into the MCF-7 cells were 0.200 ± 0.005 mM and 0.270 ± 0.025 mM, respectively. The IC_50_ of the free IFO was markedly larger than LEM-IFO and SAL-IFO when applied into MCF-7 cells (9.20 ± 2.01 mM) and HeLa cells (7.69 ± 1.88 mM). Among the tested formulas, LEM-IFO and SAL-IFO have the greatest apoptotic effect on the MCF-7 and HeLa cells, respectively. Solubilizing the IFO in the essential oils-based NE has ameliorated the antitumor efficacy of IFO.

## Introduction

Ifosfamide (IFO), a DNA- alkylating agent, induces apoptosis in the cancer cells through stimulating the caspases^1^. Usually neurotoxicity and nephrotoxicity is associated with the IFO administration^2^. Therefore, there have been many *in vitro* and *in vivo* studies on the IFO delivered in nanocarriers with the aim to eliminate its adverse side effects. IFO was encapsulated in nanostructured lipid nanoparticles^3^, poly (lactic-co-glycolic acid)-dextran polymeric nanoparticles^4^, the surface modified solid lipid nanoparticulate^5^, self-microemulsifying drug delivery systems^6^ and nano-vesicles formulated with Span 80^7^. In view of that, nanoemulsion can be one of the nanocarriers that hold a potential in solubilizing IFO and improving its bioavailability.

Nanoemulsions (NEs) are colloidal systems that consist of emulsifiers, surfactants and/or cosurfactants, oil and water^8–10^. The fractions of the NEs constituents, mainly the oil and water, control the arrangement of the surfactants in the dispersed colloidal particle. If the amount of water is more dominant, a micelle will be formed at which the oil solubilizes in the water (oil-in-water NE) but if the oil fraction exceeds the water, the micelle may get reversed and hence water-in-oil NE get produced. NEs have been always attractive for the pharmaceutical and nutraceutical industries because of their flexible structures that may solubilize both hydrophilic or hydrophobic drugs and the relative lesser amount of surfactants (<20%) used in their formation.

In order to ameliorate the properties of the NEs, they were formulated from the essential oils (ESSOs) which were found to have anticancer activities against various kinds of cancer^11,12^. Although ESSOs cannot be used as a substituent to the chemotherapy and radiotherapy, they may be combined with anticancer agents in NE formula and thereby potentiate the drug efficacy and reduce its adverse side effects. Plants containing LEM oil in its constituents were reported to have apoptotic effect in colon cancer cells^13^, human prostate carcinoma, lung carcinoma, and breast cancer cells^14^ and human cervical adenocarcinoma cells^15^. In addition, salvia oil-bearing plants were found to have cytotoxic effect in hepatoma G2 and Caco-2 cells^16^, human melanoma cells^17^, isogenic colon cancer cells^18^, breast cancer cells, amelanotic melanoma, renal cell adenocarcinoma and hormone dependent prostrate carcinoma cells^19^.

In the current study, two NE formulations were produced based on SAL and LEM oils in order to evaluate their antiproliferation activities in MCF-7 breast cancer cells and HeLa cervical cancers cells.

## Materials and Methods

### Chemicals

Ifosfamide was purchased from Baxter, US. Span 20 and Tween 80 were obtained from Sigma (Missouri, US). The 3(4,5dimethylthiazole-2-yl)-2,5-diphyneltetrazolium bromide (MTT), dimethyl sulfoxide (DMSO) and Coomassie brilliant blue were obtained from biomatik (Ontario, Canada). Ethanol and formaldehyde were purchased from Fisher Chemical (UK). The heat inactivated fetal bovine serum (FBS), and phosphate buffered saline (PBS, pH 7, 10 mM) were obtained from Lonza Walkersville (USA). Dulbeccos modified eagle medium (DMEM), trypsin and penicillin streptomycin antibiotic were obtained from Gibco life technologies (New York, US). The 4′,6-diamidino-2-phenylindole (DAPI) dihydrochloride was purchased from Invitrogen life technologies (New York, US).

### Cell lines

The human cervical cancer cell line (HeLa), human breast adenocarcinoma cell line (MCF-7) were procured from American Type Tissue Culture Collection (Manassas, VA, USA).

### Construction of the phase diagrams for the NE preparations

Two pseudo ternary phase diagrams were constructed at different weight fractions of LEM or SAL oil, water, and a surfactant mixture blended at a fixed ratio of 2:1 of Tween 80 to Span 20, respectively. They were constructed to determine the emulsion (EM) regions at which the resulted formulas may get converted to NE. The oil-in-water (O/W) NE formulas (LEM-NE and SAL-NE) were produced by selecting one of the formed EM formulas, which consisted of 5.5% (wt/wt) of surfactant mixture, 1.8% (wt/wt) LEM or SAL oil and 92.7% (wt/wt) water, followed by vortexing above 70 °C until it become clear and transparent. Three replicates for the phase diagrams and ten replicates for NE formulas were performed. The stock solution of 19 mM of IFO was prepared by directly dissolving 5 mg of IFO in 1 ml of distilled water (IFO), 1 ml of NE-based SAL oil (SAL-IFO) or 1 ml of NE-based LEM oil (LEM-IFO).

### Physical characterization of NE formulas

A key distinctive property of NE is its nanoscale particle size. The analysis of the nanodroplets of the produced NE was performed by Zetasizer (Malvern Instruments, Malvern, UK). The sizes and charges of the NE dispersed nanodroplets were expressed as z-average diameters and zeta potentials, respectively. Measurements were performed three times at 25 °C.

### Cell culture

Cells were cultured in a 25 cm^2^ cell culture flask, containing DMEM, 10% (v/v) FBS and 1% (v/v) penicillin-streptomycin, and incubated in a 5% CO_2_/95% humidified atmosphere at 37 °C. The media were changed every 48 h until confluence followed by washing with 2 ml of PBS, detachment by adding 2 ml of trypsin, and incubation at 37 °C.

### Cytotoxicity screening using MTT assay

The toxicity of the chemotherapeutic agents against the cancerous cells is evaluated by the MTT assay. A 100 µl of culture media containing 10,000 cells, counted using a countess automated cell counter (Invitrogen, US), was seeded in each well of a 96-well plate and was incubated overnight at 37 °C in a CO_2_ incubator for cell attachment. Then, cells were treated with 100 µl of different formulas, followed by incubation at 37 °C in a CO_2_ incubator for 24 h. After that, a 5 µl of MTT reagent was added to each well, mixed gently for one minute and incubated for 3 to 4 h at 37 °C in a CO_2_ incubator. Then, the culture medium containing MTT reagent was removed, followed by adding a 100 µl of DMSO and incubation for 2 h. Each tested concentration for all formulas ware repeated three times. The Absorbance (Abs) was read at 540 nm using a microplate reader (BioTek, US). Wells, included culture media, were considered negative control while culture media containing cells served as a positive control. The percentages of cell viabilities were determined by the following equation:

$${\rm{Cell}}\,\mathrm{viability}( \% )=\frac{({\rm{Abs}}\,{\rm{of}}\,{\rm{treated}}\,{\rm{cells}}-{\rm{Abs}}\,{\rm{of}}\,{\rm{negative}}\,{\rm{control}})}{({\rm{Abs}}\,{\rm{of}}\,{\rm{positive}}\,{\rm{control}}-{\rm{Abs}}\,{\rm{of}}\,{\rm{negative}}\,{\rm{control}})}\times 100$$

### Visualization of cell death by the light microscopy

The HeLa and MCF-7 cell deaths were observed under the phase contrast inverted microscope (Olympus 1 × 51, Japan). Cells were counted and plated at a density of 10,000 cells per well into 96-well plate containing 100 μl of growth media in each well. Then, they were incubated with 100 μl of the desired drug formula for 24 h followed by washing twice with 100 µl of PBS and fixation by the addition of 100 µl of 4% formaldehyde for 5 min. After that, the fixation solution was discarded and the cells were washed with 100 µl of PBS and stained with 100 µl of 10% Coomassie blue for 10 min. Then, the stain solution was discarded, washed with 100 µl of distilled water twice and left to dry at room temperature, 25 °C. The experiments were performed three times.

### Apoptosis detection by Nuclear DNA staining with DAPI

The nuclear condensations of the treated cells undergoing apoptosis were detected using the DAPI stain which is able to permeate the cell undergoing apoptosis and get attached to A-T rich regions in DNA to give a strong blue fluorescent dye. Cells were counted and plated at a density of 5 × 10^4^ cells per 500 μl of growth media in each well of 24 well-plates. Cells, treated with 500 µl of the desired formula, were incubated for 24 h. Then, cells were equilibrated with 300 µl of PBS, fixed by 200 µl of formaldehyde and stained with 300 µl of 300 nM of DAPI solution and incubated for 1–5 min at 25 °C. Triplicate experiments were implemented. The cells were observed by the inverted fluorescent microscope (Leica CRT6000, Germany). The percentages of fluorescent intensity were estimated through using Image J 1.43 n software (Rasband, W.S., ImageJ, National Institutes of Health, US).

### Statistical analysis

Statistical analyses for testing the differences between multiple samples were implemented by one-way analysis of variance (ANOVA) based on F-test used for assessing the equality of variance and the skewness test for evaluating the normal distribution of the samples data. Following ANOVA F-test, the post hoc analyses between the two samples were performed by measuring the p-values for the pairwise using the Tukey’s simultaneous comparison t-values. MegaStat Excel (version 10.3, Butler University, Indianapolis, IN) was used for all of the statistical analyses. The statistical discrepancies between the samples were considered when P-value < 0.05.

## Results

### Pseudo ternary phase diagrams

**Figure 1:**
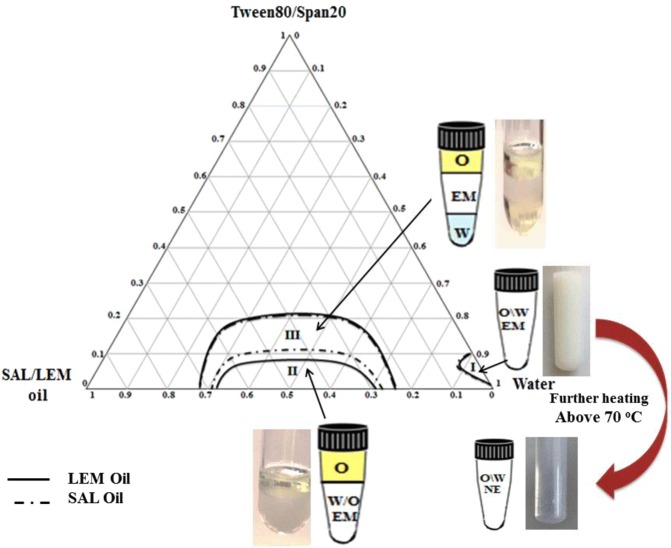
Pseudo ternary phase diagrams constructed at different weight fractions of lemon oil () or salvia oil (), water, and a surfactant mixture blended at a fixed ratio of 2:1 of Tween 80 to Span 20, respectively.

As exhibited in Fig. 1, the three phase region of the LEM oil was larger than SAL oil region whereas the two phase region for LEM oil was smaller than SAL oil, although the location of the two and three phases in the bottom of the phase diagrams for LEM and SAL oils were similar. The one phase regions of the O/W EM of both of LEM and SAL oils were located at the corner at which the water fraction was greater than the oil fraction and the Tween 80/Span 20 fractions. The EM solution for the selected oil, consisted of 1.8% oil, 5.5% Tween 80/Span 20 and 92.7% water, was converted to NE by heating and vortexing the solution above 70°C for one hour.

### Physical characteristics of ESSO-based NE

According to Table 1, the z-average diameters of LEM-NE and SAL-NE did not markedly change when loaded with IFO. However, the absolute value of the negative zeta potential of LEM-NE was less than LEM-IFO whereas the zeta potential of SAL-NE was greater than SAL-IFO. It is worth mentioning that SAL-IFO has the largest z-average diameter and the smallest absolute value of the negative zeta potential among the tested NE formulas. The sizes of the dispersed nanodroplets of the entire NE formulas were homogeneously distributed as the polydispersity indexes (PDIs), measured through dividing the standard deviation by the mean of the z-average diameter, were less than 0.250.

**Table 1:** The physical characteristics of the NE-based ESSO measured by zetasizer for three determinations.

Formula	Zeta Potential (mV)		Range of z-average diameter (nm)		PDI
LEM-NE	−3.29 ± 0.59	HS	40.72–58.26	NS	0.177
LEM-IFO	−6.035 ± 0.77		49.15–61.81		0.114
SAL- NE	−7.9 ± 0.11	HS	52.12–56.18	NS	0.038
SAL-IFO	−1.14 ± 0.08		56.64–64.62		0.066

PDI: is the poly dispersity index; HS: very highly significant difference between the tested formula (P < 0.001); NS: no significant difference between the tested formula (P > 0.05).

### Determination of cell viability using the MTT assay

**Figure 2:**
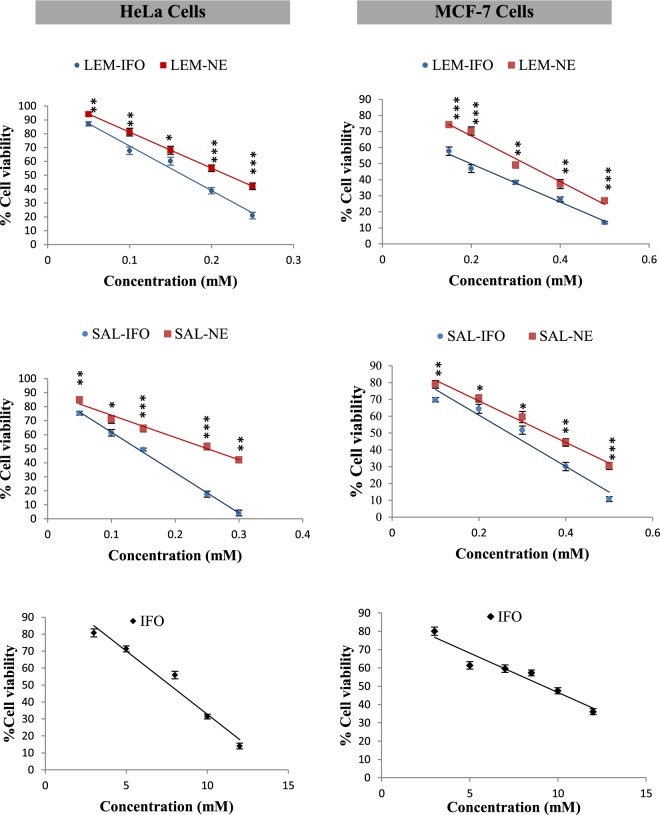
The percentages of cell viability of HeLa and MCF-7 cells treated for 24 h at different concentration of the tested formulas. *Error bars* represent the standard deviation for n = 3. The levels of the differences between the formulas at each concentration are ranked as very highly (***P < 0.001), highly (**0.001 ≤ P < 0.01) and (*0.01 ≤ P < 0.05) significant.

To investigate the cytotoxicity effect of NE formulations on the HeLa and MCF-7 cells, they were treated with different concentration of LEM-NE, LEM-IFO, SAL-NE, SAL-IFO and IFO solution for 24 h. As displayed in Fig. 2, there was a steady decline in the HeLa and MCF-7cells viabilities as the concentration of the subjected formulas increase. However, the viabilities of HeLa cells show sharp drop with increase concentration whereas the viabilities of MCF-7 cells were gradually decreased when subjected into the tested formula.

In terms of the cancer cells growth inhibition, it has been found that the IC_50_’s of all of the tested NE formulas were markedly less than the IC_50_’s of IFO, which were 7.69 ± 1.88 and 9.20 ± 2.01 mM when applied into HeLa and MCF-7 cells, respectively (P < 0.001). Among the drug-free NE formulas, the IC_50_ of LEM-NE (0.219 ± 0.005 mM) was comparable to the IC_50_ of SAL-NE (0.250 ± 0.001 mM) when applied into the HeLa cells. In contrast, the IC_50_ of LEM-NE (0.321 ± 0.015 mM) was considerably less than the IC_50_ of SAL-NE (0.355 ± 0.005 mM) (P < 0.001) when subjected into the MCF-7 cells. Regarding the NE-loaded IFO formulas, the comparable IC_50_’s of LEM-IFO (0.165 ± 0.025 mM) and SAL-IFO (0.141 ± 0.035 mM), applied into HeLa cells, were significantly less than the IC_50_’s of LEM-IFO (0.200 ± 0.005 mM) and SAL-IFO (0.270 ± 0.025 mM), subjected into MCF-7 cells (P < 0.001).

### Morphological changes of HeLa and MCF-7 cells

**Figure 3:**
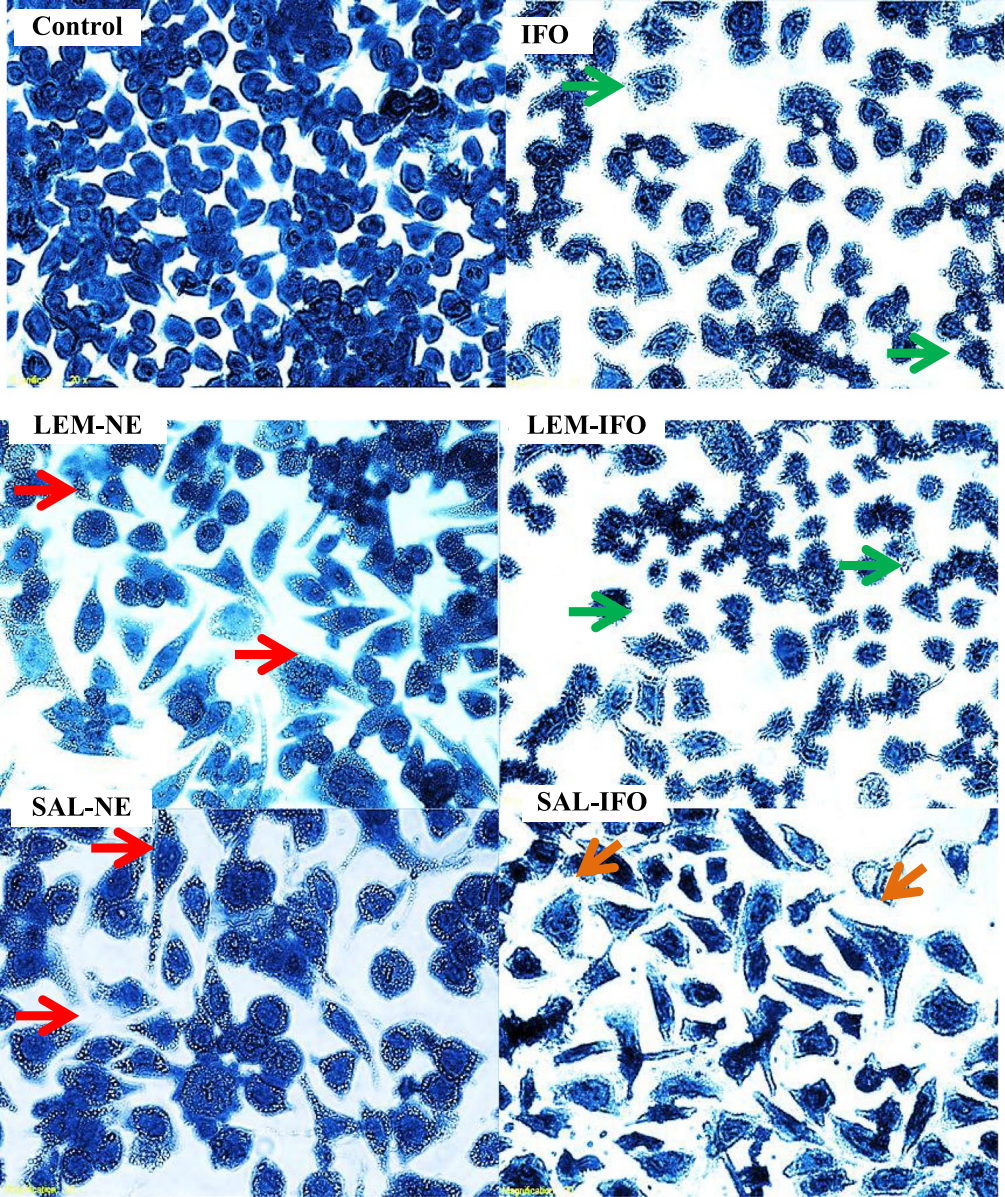
Light Microscopy images of HeLa cells subjected into different formulas for 24 h at their IC_50_s (n = 3). Images were magnified at 400x. The arrows colored green, orange and red displayed the membrane blebbing with collapse nucleus, the enlarged cells and the digestive vesicles, respectively.

**Figure 4:**
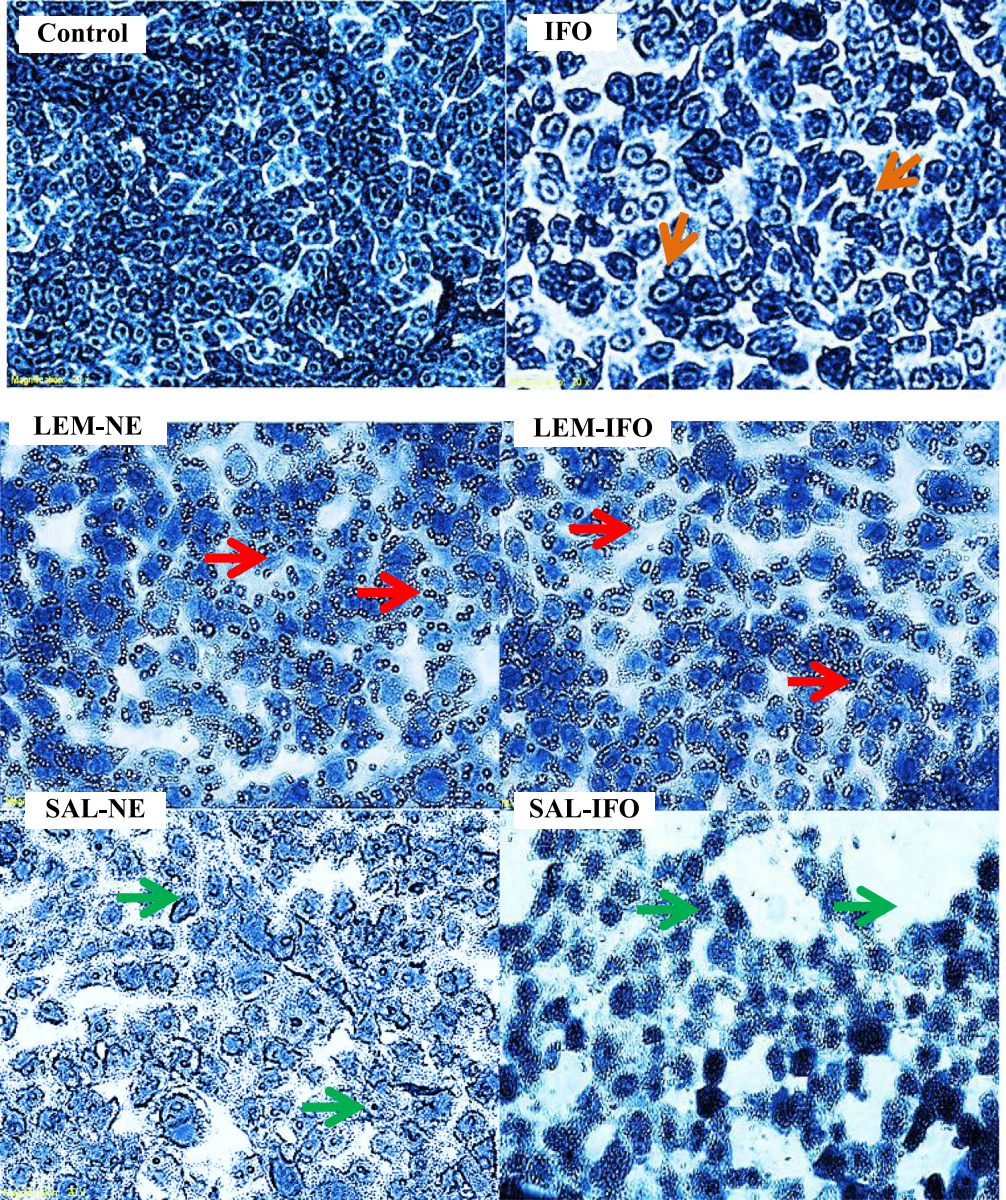
Light Microscopy images of MCF-7 cells subjected into different formulas for 24 h at their IC_50_s (n = 3). Images were magnified at 400x. The arrows colored orange, red and green represented condensed chromatin, extracellular apoptotic bodies and ghost cells, respectively.

As shown in Figs 3 and 4, HeLa and MCF-7 cells exhibited morphological changes after treatment for 24 h at the IC_50_’s of the tested formulas. All of the treated HeLa cells have endured different stages of apoptosis (Fig. 3). Cells treated with IFO and LEM-IFO have revealed late signs of apoptosis as their size has shrunk and membrane blebbing with collapse nucleus were seen whereas cells treated with SAL-IFO were enlarged and their shape were altered. In contrast, LEM-NE and SAL-NE subjected into the HeLa cells have stimulated the formation of the digestive vesicles and apoptotic bodies. It should be noted that intercellular spaces between cells were displayed in all of cells treated with tested formulas.

Regarding the MCF-7 cells (Fig. 4), LEM-NE and LEM-IFO have markedly induced the formation of the extracellular apoptotic bodies. Cells treated with SAL-NE and SAL-IFO have endured late sign of apoptosis as ghost cells that lack nucleus were seen. On the other hand, cells treated with free IFO have revealed condensed chromatid without any alteration in their shape.

### Nuclear morphological change

**Figure 5:**
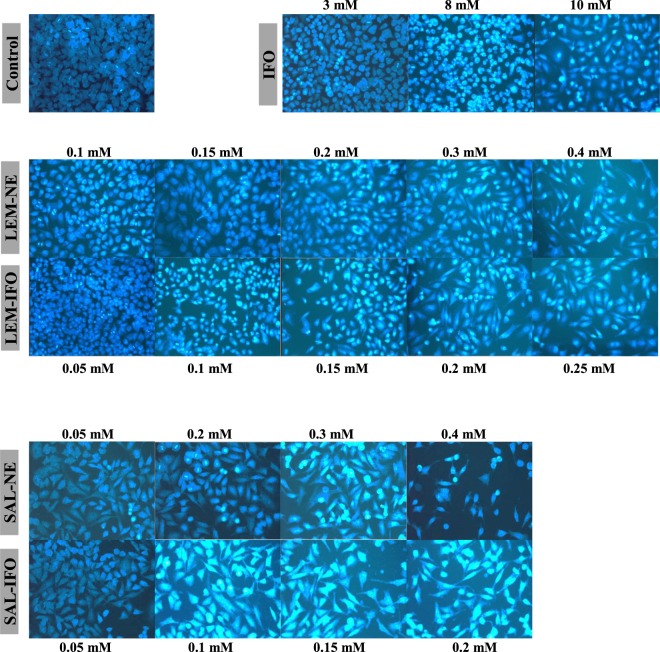
The nuclear morphology changes of HeLa cells stained with DAPI and treated with different concentrations of the drug formulas (n = 3) for 24 h.

**Figure 6:**
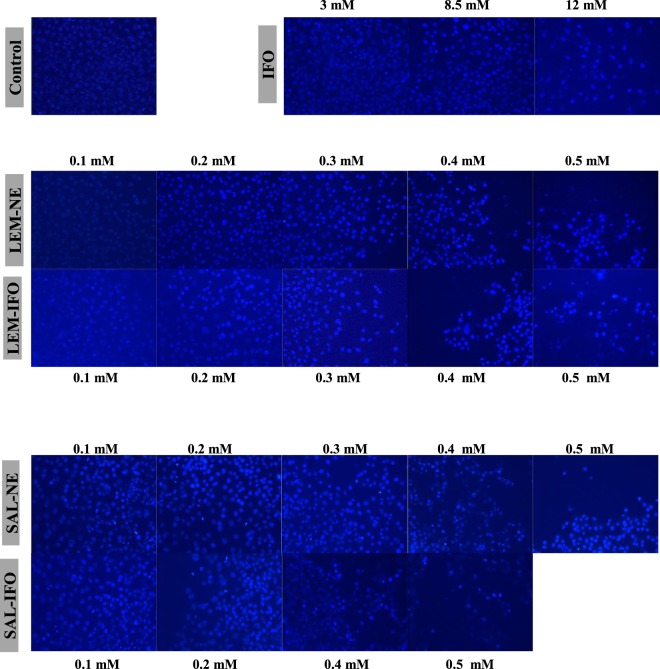
The nuclear morphology changes of MCF-7 cells stained with DAPI and treated with different concentrations of the drug formulas (n = 3) for 24 h.

**Figure 7:**
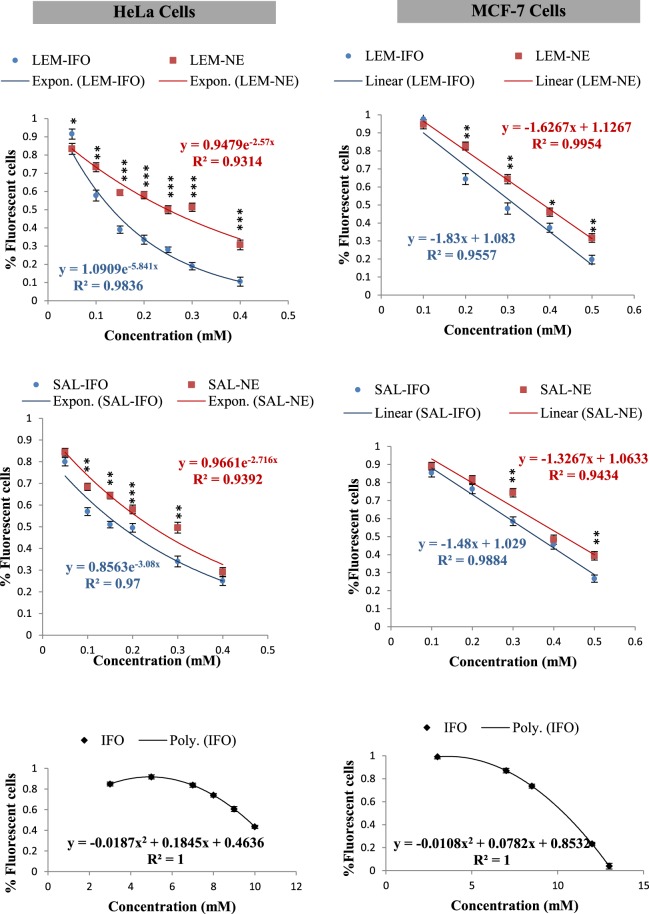
The cell counts at different concentrations of the tested formulas measured from the fluorescent microscopic images stained with DAPI (Figs 5 and 6). *Error bars* represent the standard deviation for n = 3. The levels of the differences between the formulas at each concentration are ranked as very highly (***P < 0.001), highly (**0.001 ≤ P < 0.01)) and (*0.01 ≤ P < 0.05) significant.

To determine the cell death due to apoptosis, DAPI stain was utilized to fluoresce the altered nuclei. As shown in Figs 5, 6 and 7, all of the tested formulas have decreased the cell counts as their concentrations have increased when subjected into either HeLa or MCF-7 cells. However, the shapes of the curves have differed. The counts of MCF-7 cells have declined linearly while the counts of HeLa cells have decreased exponentially when treated with the drug-free NEs and drug-loaded NEs (Fig. 7). In contrast, the increase in the IFO concentrations has caused a parabolic decrease in both of MCF-7 and HeLa cells counts. Interestingly, the decrease in both of MCF-7 and HeLa cells subjected into the drug-loaded NE was higher relative to the cells treated with drug-free NE.

## Discussion

Nanocarriers, like NEs, can be involved in combining two or more anticancer agents with different physical properties in order to assure their simultaneous delivery into the target cells and thereby a synergistic effect of the incorporated agents may develop. Compared to the IC_50_ of IFO, the IC_50_’s of the entire NE formulas were markedly reduced in both of MCF-7 and HeLa cells which can be attributed to the small sizes and negative zeta potentials of the NEs nanodroplets that facilitate their permeation into the cancer cells^20^. Additionally, the incorporation of the IFO and ESSOs, SAL and LEM oils, into the NEs may enhance their accumulation in the cancer cells and thereby potentiate their anticancer activity^21^. Interestingly, the produced nanoemulsions formulations in the present study have the narrowest average size as compared with the previous studies^3–7^. In fact, the IC_50_’s subjected to HeLa cells of the SAL-IFO and LEM-IFO was reduced three times relative to the formulation produced in previous work at self-microemulsifying drug delivery systems^6^.

The response of the MCF-7 cells to the NE formulas has differed from the HeLa cells. In general, HeLa cells were more sensitive than the MCF-7 cells as the IC_50_’s of the tested formulas subjected into the HeLa cells were less than the IC_50_s administered into the MCF-7 cells. The growth of the MCF-7 cells was greatly impeded when subjected into the LEM-IFO meanwhile SAL-IFO has the best inhibition effect on the growth of the HeLa cells. The great apoptotic effect of LEM-IFO on the MCF-7 cells may stem from the extracellular vesicle formation, seen under the light microscope, indicating that LEM-IFO has got attached into the cell membrane and induced apoptosis integrated with autophagocytosis^22^. In contrast, the light microscopy images revealed that the apoptotic effect of SAL-IFO on the HeLa cells has enlarged the cancer cells and damaged the nucleus without vesicle formation which implies that SAL-IFO has passed the cell membrane. Although both of LEM and SAL oils have antiproliferation activity, they have different mechanism of action in inducing cell apoptosis. LEM oil was found to impede the growth of the colon cancer cells through suppressing PI3K/Akt pathway that lead to the mitochondrial death^13^. On the other hand, many research studies have reported the antiangiogenic activity of SAL oil that prevents the tumor growth and invasion in various types of cancers^16–19,23^.

According to the analysis of the DAPI images, the growth of the MCF-7 cells was linearly decreased whereas the growth of the HeLa cells was exponentially decreased as the concentration of the subjected NE increased. This inverse relation between the growth of the cells and the NE concentration was first-order reaction which means that the inhibition process relies on one component. In other words, the nanodroplets of the NE formulas did not get degraded in the cancer cell and may act as a one metabolite. In addition, the inhibition of the LEM-IFO and SAL-IFO has reduced the growth of the MCF-7 and HeLa cells with similar trends when compared to the LEM-NE and SAL-NE. This implies that there has been a synergistic effect caused by the incorporation of the IFO into the ESSOs-based NE^24^. On the other hand, the free IFO applied into either MCF-7 or HeLa cells has caused a parabolic decrease in the growth of the cancer cells as its concentration increase indicating that the inhibition process is a second order reaction which depends on two metabolites. In fact, IFO may get degraded in the cell and form the two active metabolites, IFO mustard and choroacetaldehyde, that may induce apoptosis in the cancer cells^25^.

## Conclusion

Incorporating the IFO into the ESSOs-based NE has markedly improved its cytotoxicity in the HeLa and MCF-7 cells. HeLa cells were more sensitive to the tested formulas than the MCF-7 cells. Among the NE formulas, LEM-IFO has the best apoptotic and inhibition effect on the MCF-7 cells while SAL-IFO has the best cytotoxic effect on the HeLa cells. Further studies have to be performed on the mechanism of apoptosis of LEM-IFO and SAL-IFO.
